# Peer-provided psychological intervention for Syrian refugees: results of a randomised controlled trial on the effectiveness of Problem Management Plus

**DOI:** 10.1136/bmjment-2022-300637

**Published:** 2023-02-08

**Authors:** Anne M de Graaff, Pim Cuijpers, Jos W R Twisk, Barbara Kieft, Sam Hunaidy, Mariam Elsawy, Noer Gorgis, Theo K Bouman, Miriam J J Lommen, Ceren Acarturk, Richard Bryant, Sebastian Burchert, Katie S Dawson, Daniela C Fuhr, Pernille Hansen, Mark Jordans, Christine Knaevelsrud, David McDaid, Naser Morina, Hanspeter Moergeli, A-La Park, Bayard Roberts, Peter Ventevogel, Nana Wiedemann, Aniek Woodward, Marit Sijbrandij

**Affiliations:** 1 Clinical, Neuro, and Developmental Psychology, Amsterdam Public Health Research Institute, Vrije Universiteit Amsterdam, Amsterdam, Noord-Holland, The Netherlands; 2 International Institute for Psychotherapy, Babeș-Bolyai University, Cluj-Napoca, Romania; 3 Department of Epidemiology and Data Science, VU University Medical Centre Amsterdam, Amsterdam, Noord-Holland, The Netherlands; 4 ARQ National Psychotrauma Centre, Amsterdam, The Netherlands; 5 i-Psy, Parnassia Groep, The Hague, South Holland, The Netherlands; 6 Clinical Psychology and Experimental Psychopathology, University of Groningen, Groningen, The Netherlands; 7 Department of Psychology, Koc Universitesi, Istanbul, Turkey; 8 School of Psychology, UNSW Sydney, Sydney, New South Wales, Australia; 9 Department of Education and Psychology, Freie Universität Berlin, Berlin, Germany; 10 Health Services Research Unit, London School of Hygiene and Tropical Medicine, London, UK; 11 Health Sciences, University of Bremen, Bremen, Bremen, Germany; 12 Department of Prevention and Evaluation, Leibniz Institute for Prevention Research and Epidemiology-BIPS, Bremen, Germany; 13 Reference Centre for Psychosocial Support, International Federation of Red Cross and Red Crescent Societies, Copenhagen, Denmark; 14 Research and Development Department, War Child Holland, Amsterdam, The Netherlands; 15 Amsterdam Institute of Social Science Research, University of Amsterdam, Amsterdam, Noord-Holland, The Netherlands; 16 Department of Health Policy, The London School of Economics and Political Science, London, UK; 17 Department of Consultation-Liaison Psychiatry and Psychosomatic Medicine, University Hospital Zurich, Zurich, Switzerland; 18 Public Health Section, UNHCR, Geneva, Switzerland; 19 KIT Health, KIT Royal Tropical Institute, Amsterdam, Noord-Holland, The Netherlands

**Keywords:** Depression & mood disorders, Anxiety disorders, Adult psychiatry

## Abstract

**Background:**

The mental health burden among refugees in high-income countries (HICs) is high, whereas access to mental healthcare can be limited.

**Objective:**

To examine the effectiveness of a peer-provided psychological intervention (Problem Management Plus; PM+) in reducing symptoms of common mental disorders (CMDs) among Syrian refugees in the Netherlands.

**Methods:**

We conducted a single-blind, randomised controlled trial among adult Syrian refugees recruited in March 2019–December 2021 (No. NTR7552). Individuals with psychological distress (Kessler Psychological Distress Scale (K10) >15) and functional impairment (WHO Disability Assessment Schedule (WHODAS 2.0) >16) were allocated to PM+ in addition to care as usual (PM+/CAU) or CAU only. Participants were reassessed at 1-week and 3-month follow-up. Primary outcome was depression/anxiety combined (Hopkins Symptom Checklist; HSCL-25) at 3-month follow-up. Secondary outcomes included depression (HSCL-25), anxiety (HSCL-25), post-traumatic stress disorder (PTSD) symptoms (PTSD Checklist for Diagnostic and Statistical Manual of Mental Disorders, Fifth Edition; PCL-5), impairment (WHODAS 2.0) and self-identified problems (PSYCHLOPS; Psychological Outcomes Profiles). Primary analysis was intention-to-treat.

**Findings:**

Participants (n=206; mean age=37 years, 62% men) were randomised into PM+/CAU (n=103) or CAU (n=103). At 3-month follow-up, PM+/CAU had greater reductions on depression/anxiety relative to CAU (mean difference −0.25; 95% CI −0.385 to −0.122; p=0.0001, Cohen’s *d*=0.41). PM+/CAU also showed greater reductions on depression (p=0.0002, Cohen’s *d*=0.42), anxiety (p=0.001, Cohen’s *d*=0.27), PTSD symptoms (p=0.0005, Cohen’s *d*=0.39) and self-identified problems (p=0.03, Cohen’s *d*=0.26), but not on impairment (p=0.084, Cohen’s *d*=0.21).

**Conclusions:**

PM+ effectively reduces symptoms of CMDs among Syrian refugees. A strength was high retention at follow-up. Generalisability is limited by predominantly including refugees with a resident permit.

**Clinical implications:**

Peer-provided psychological interventions should be considered for scale-up in HICs.

**WHAT IS ALREADY KNOWN ABOUT THIS TOPIC**

- Common mental disorders are highly prevalent among refugee populations.
- Problem Management Plus (PM+) is a non-specialist-delivered intervention that is effective in reducing symptoms of common mental disorders in communities affected by adversity in low- and middle-income countries.

**WHAT THIS STUDY ADDS**

- This study shows that PM+ is effective in improving symptoms of depression and anxiety in refugees in a high-income setting.
- PM+ also improves symptoms of post-traumatic stress disorder, daily functioning and self-identified problems.

**HOW THIS STUDY MIGHT AFFECT RESEARCH, PRACTICE, OR POLICY**

- Non-specialist-delivered interventions should be considered for scaling up in refugee populations in high-income settings.

## Introduction

The war in Syria has led to an unprecedented number of forcibly displaced people. Almost 7 million refugees have sought refuge primarily in neighbouring countries as well as in Europe.^1^ Exposure to severe stressors, such as violence, detention and lack of basic needs have been widely reported.^2^ After migration, refugees may continue to experience hardships such as lengthy asylum procedures, financial insecurity and social isolation.^3^ The types of hardships may vary between refugees in high-income countries (HICs) versus low-/middle-income countries (LMICs). Prominent stressors reported by Syrian refugees/asylum seekers in Switzerland included concerns about employment and housing, whereas concerns about finances (Türkiye) and living conditions (refugee camp Jordan) were more prominent in LMICs.^4^ These stressors can cause a significant psychological burden on individuals. Meta-analytic evidence of common mental disorders (CMDs) among refugees/asylum seekers show rates as high as 32% for depression and 31% for post-traumatic stress disorder (PTSD).^5^ Prevalence rates among Syrian refugees in European settings, such as Sweden, were 40% and 30%, respectively.^6^ Although (specialist) mental health services are available in HICs such as the Netherlands, refugees/asylum seekers may not access them due to several barriers including waitlists, stigma and communication difficulties.^7^

To improve the access to evidence-based psychological interventions in underserved communities, the WHO developed a series of scalable interventions. One of these is Problem Management Plus (PM+), developed to target depression, anxiety and general distress in communities affected by adversity.^8^ PM+ is potentially scalable due to its brevity (few sessions), transdiagnostic target (aiming at a range of symptoms instead of single disorders), task-sharing approach (delivery by non-specialist helpers without formal psychotherapy training) and potential cost-effectiveness.^9^ Earlier studies on PM+ in non-refugee samples in Pakistan and Kenya showed its effectiveness in reducing depression, anxiety, PTSD, functional impairment and self-identified problems.^10 11^

The STRENGTHS consortium investigates the effectiveness, cost-effectiveness and implementation of PM+ for Syrian refugees in countries in Europe and the Middle East.^12^ A group version of PM+ has been evaluated among Syrian parents in a Jordanian refugee camp, with beneficial effects on depression, self-identified problems and disciplinary parenting, but not on anxiety, PTSD or functioning.^13^ No study has yet investigated the effectiveness of PM+ for refugees in a HIC. In August 2022, the Netherlands registered 45 750 Syrian asylum seekers/refugees.^14^ A pilot study on individual PM+ among 60 Syrian refugees in the Netherlands showed acceptability and feasibility in a high-income setting and suggested it might be effective in reducing symptoms of CMDs.^15^

This study aimed to evaluate the effectiveness of PM+ on symptoms of depression/anxiety (total score; primary outcome) and on depression, anxiety, symptoms of PTSD, functional impairment and self-identified problems among Syrian refugees in the Netherlands.

## Methods

### Design

This single-blind randomised controlled trial (RCT) was conducted by Vrije Universiteit Amsterdam (VU) in collaboration with i-Psy mental healthcare. The trial was approved by the Research Ethics Review Committee at VU Medical Center (NL61361.029.17)^16^ and prospectively registered in the Netherlands Trial Registry (No 7552). The CONSORT checklist is supplements (online supplemental file 1).

10.1136/bmjment-2022-300637.supp1 Supplementary data

### Procedures

Adult (18 years or above) Arabic-speaking Syrian refugees were recruited through community centres, non-governmental organisations, reception centres, language schools and social media. With ‘Syrian refugees’ we refer to individuals from Syria who requested asylum after the start of the war in 2011 regardless of current resident status. Oral and written informed consent (IC) was obtained from all participants before screening. Participants were included if they reported elevated levels of psychological distress (Kessler Psychological Distress Scale; K10 >15)^17^ and impaired daily functioning (WHO Disability Assessment Schedule; WHODAS 2.0 >16).^18^ Participants were excluded and referred to the general practitioner/specialist services if they met any of the following criteria: acute medical conditions, imminent suicide risk (PM+ manual suicidality assessment), expressed acute needs/protection risks, indications of severe mental disorders (eg, psychotic disorders) or cognitive impairment (eg, severe intellectual disability; PM+ manual observation checklist). Participants were also excluded if they received ongoing treatment in specialised mental healthcare to prevent potential interference between the ongoing treatment and PM+.

The baseline assessment included questionnaires on demographics, clinical outcomes, daily functioning, stressful events and health service utilisation (reported elsewhere). Participants were reassessed 1 week and 3 months after the intervention (ie, 6 weeks and 4.5 months after baseline). Assessments were conducted in the online questionnaire tool Survalyzer. For each assessment, participants were contacted by an Arabic-speaking assessor who sent a secured online link for the self-report questionnaires, conducted a brief phone-based interview on health service utilisation and assisted in case of lower literacy. Participants were remunerated €8.50 for each follow-up assessment. Assessors had at least a university degree and were trained on questionnaire administration, general interview techniques, CMDs, psychological first aid and research ethics. Serious adverse events (SAEs) were recorded and monitored throughout the study.

After baseline, participants were randomised 1:1 into PM+ in addition to care as usual (PM+/CAU) or CAU alone. A randomisation list with permuted block sizes 4-6-8 was generated in R^19^ by an independent researcher not involved in the rest of the study. A researcher not involved in the outcome assessments informed participants about allocation using sealed opaque envelopes. Outcome assessors were masked to group allocation. To evaluate the success of masking, assessors indicated after each assessment whether group allocation was revealed.

### Study arms

#### Problem Management Plus

PM+ consists of five 90-min, weekly in-person sessions with a non-specialist helper.^8^ It integrates four evidence-based behavioural strategies: stress management using diaphragmatic breathing (session 1), problem-solving (session 2), behavioural activation by re-engaging with pleasant/task-oriented activities (session 3) and accessing social support (session 4). Homework practice is scheduled following each session and reviewed in the next session. Psychoeducation is delivered in session 1 and relapse prevention in session 5. Helpers were Arabic (and Dutch or English) speaking Syrian refugees with at least high school education and (professional) background in education, social work or related field and a Certificate of Conduct. Helpers received an 8-day training on CMDs, basic counselling skills, delivery of intervention strategies and self-care, followed by a practice case. Helpers met weekly for group supervision by a PM+ supervisor. PM+ trainers/supervisors were mental health professionals from i-Psy, VU and University of Groningen who had received a 5-day training covering elements of training of helpers and training/supervision skills. Due to COVID-19 restrictive measures (the first partial lockdown in March 2020), participants were given the option for in-person or video call sessions.

To evaluate treatment fidelity, helpers completed a checklist addressing requisite PM+ components for each session. Additionally, all PM+ participants were asked IC to audio record sessions for independent assessment of fidelity. Two assessors (ME/SH) with knowledge of the PM+ manual independently rated a random sample of 10 tapes per session (50 in total) using the PM+ checklist for adequate delivery of treatment elements (yes/no).^15^

PM+ and other interventions investigated in STRENGTHS were adapted for use in Syrian refugee populations.^12^ The full process was coordinated by the IFRC Psychosocial Centre in eight countries and included literature review, stakeholder engagement, rapid qualitative assessments (n=450 respondents, of which 361 with a Syrian refugee background),^20^ literal translation, cognitive interviews (n=30 respondents, of which 24 with a Syrian refugee background), adaptation workshops and finalisation of the manuals. The adaptations and their justifications were captured using a framework for the adaptation of psychological interventions.^21^ Generally, core components such as the therapeutic strategies (eg, diaphragmatic breathing) were retained, while case examples were rephrased to be relevant for Syrian refugees.

#### Care as usual

CAU includes all (mental) health services ranging from primary to specialist mental healthcare that refugees may access in the Netherlands. For participants without a residence permit residing in a reception centre, the Central Agency for the Reception of Asylum Seekers contracted a primary care provider for on-site mental health services (eg, psychological counselling) or referral to external specialist services. Participants resettled in the community (with residence permit/Dutch nationality) pay mandatory basic health insurance and can access mental health services via their local general practitioner.^22^

#### Measures

The primary outcome concerned symptoms of depression/anxiety assessed with the 25-item Hopkins Symptom Checklist (HSCL-25).^23^ We used item mean scores (range 1–4) for both total scale (primary outcome) and subscales (secondary outcomes) in the analyses. To differentiate between individuals with or without probable depression/anxiety, we used a validated cut-off score of 2.10 for depression and 2.00 for anxiety.^23^

Secondary outcomes included the 12-item WHODAS 2.0^18^ to measure functional impairment. Items were rated on a 1–5 scale (total range 12–60). Sociodemographic information was collected using the demographic section (adapted) of the WHODAS 2.0^18^ and included gender, age, living situation, education, marital status, work status, refugee status and time of displacement. PTSD symptoms were assessed using the 20-item PTSD Checklist for Diagnostic and Statistical Manual of Mental Disorders, Fifth Edition (PCL-5).^24^ Items were scored on a 0–4 scale (total range 0–80). A score of 33 or higher was used as an indication of probable PTSD. Self-identified problems were assessed using the Psychological Outcomes Profiles (PSYCHLOPS) on a 0–5 scale (total range 0–20).^25^

Other measures included past and ongoing (severe) stressors. The number of traumatic events was assessed using a 27-item checklist^3^ adapted for use in the current project. Items were scored 1 (yes) or 0 (no) (total range 0–27). Seventeen post-migration living difficulties were scored on a 0–4 scale using the Post-Migration Living Difficulties checklist.^3^ Items with a score of 2 (moderately serious problem) or higher were regarded as positive responses and summed for analysis (range 0–17).

The reliabilities (Cronbach’s α) at baseline were 0.93 (HSCL-25 total), 0.90 (HSCL-25 depression), 0.87 (HSCL-25 anxiety), 0.77 (WHODAS 2.0) and 0.93 (PCL-5). Arabic translations of validated measures were identified, and if unavailable translated/back-translated.^15^

#### Analyses

Original power calculations were based on prior RCTs on PM+ in other populations^10 11^ but were adapted based on the pilot RCT among Syrian refugees in the Netherlands.^15^ The pilot RCT indicated an effect size of *d*=0.45 in reducing HSCL-25 scores,^15^ resulting in a required sample size of 64 per group (Cohen’s *d*=0.45, power=0.90, α=0.05, two-sided). Considering an expected 30% attrition at 3-month follow-up, we aimed to include 184 participants (92 in PM+/CAU and 92 in CAU).

The primary analysis was intention-to-treat. We used linear mixed models (LMMs) in R.^19^ To estimate the treatment effect on average over time, time was coded 1 for both the 1-week and 3-month follow-up assessment. To estimate treatment effects at both follow-up assessments separately, two dummy variables were used (one for the 1-week follow-up and one for the primary endpoint analysis at 3-month follow-up). For both, the interaction between condition and the time variable(s) was added to the model, which also included a random intercept on the subject level. Because condition itself is not added to the model, the intercept reflects the baseline value for both conditions and therefore the analysis is adjusted for the baseline differences between conditions.^26^ In this model, the regression coefficients of the interaction terms are the effect estimates (ie, mean difference between the two arms) at the two time points. Treatment effects were investigated for the primary outcome of depression/anxiety (HSCL-25 total score), as well as secondary outcomes (ie, depression, anxiety, functional impairment, symptoms of PTSD and self-identified problems). Covariate-adjusted LMMs were performed by adding relevant covariates measured at baseline (ie, gender, age, education, work status; number of traumatic events; post-migration living difficulties; and probable depression, anxiety and PTSD) to the above-mentioned model for the primary and secondary outcomes. These variables were also investigated as potential effect modifiers (ie, added in interaction with the condition at 1-week/3-month follow-up) to the LMM of the primary outcome. Cohen’s *d* was calculated by dividing the mean difference between the conditions by the raw pooled SD at that assessment. Sensitivity analyses were carried out including participants retained at 3-month follow-up (completers) and including only participants of the PM+/CAU group who completed at least four sessions (per protocol).

The reliable change index was calculated to evaluate whether the change scores from baseline to follow-up were reliable and clinically significant.^27^ The number needed to treat was estimated for depression and anxiety at 3-month follow-up using the delta method in logistic regression.

Across all analyses, two-tailed tests were reported where p <0.05 indicates statistical significance.

## Results

### Participants

**Figure 1:**
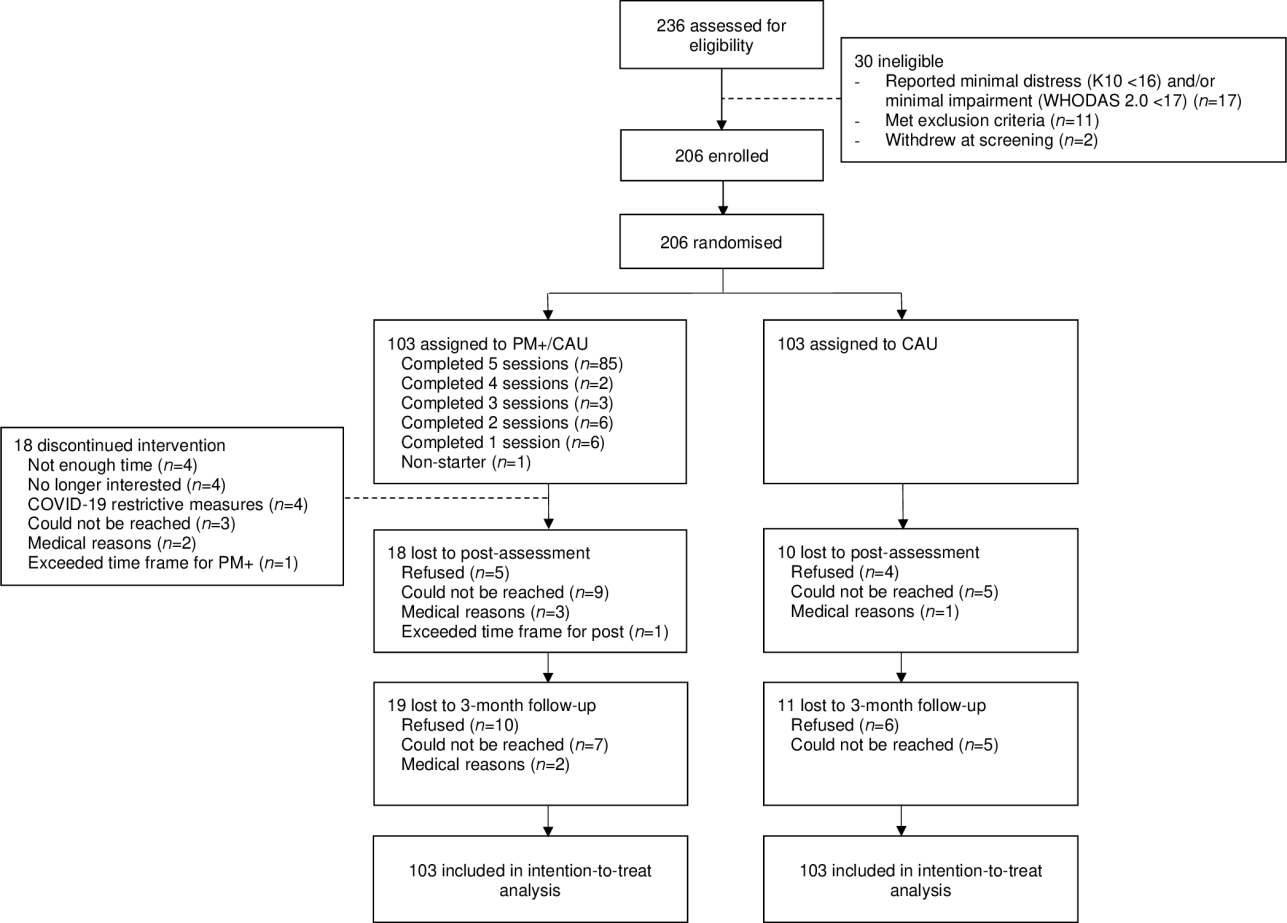
CONSORT flow diagram

Between March 2019 and December 2021, 758 individuals agreed to be contacted by VU of which 236 provided IC and completed screening. Thirty participants were excluded (see CONSORT (Consolidated Standards of Reporting Trials) flow diagram in figure 1). Of the 206 included participants, 127 (61.7%) were men, and the average age was 26.5 years (range 18–69 years, SD=11.7). Randomisation resulted in 103 participants being allocated to PM+/CAU and 103 to CAU only. Sample characteristics are presented in table 1.

**Table 1:** Baseline characteristics

	Full sample (N=206)	PM+/CAU (n=103)	CAU (n=103)
Gender, no of men (%)	127 (61.7)	73 (70.9)	54 (52.4)
Age, mean (SD) (range)	36.52 (11.72) (18–69)	36.35 (11.97) (18–69)	36.69 (11.52) (19–67)
Marital status, n (%)			
Never married	70 (34.0)	38 (36.9)	32 (31.1)
Currently married	99 (48.1)	51 (49.5)	48 (46.6)
Separated	4 (1.9)	2 (1.9)	2 (1.9)
Divorced	24 (11.7)	8 (7.8)	16 (15.5)
Widowed	5 (2.4)	2 (1.9)	3 (2.9)
Cohabiting	4 (1.9)	2 (1.9)	2 (1.9)
Work status			
Paid work	36 (17.5)	15 (14.6)	21 (20.4)
Non-paid work	30 (13.6)	17 (16.5)	13 (12.6)
Keeping house	7 (3.4)	5 (4.9)	2 (1.9)
Retired	2 (1.0)	1 (1.0)	1 (1.0)
Unemployed	40 (19.4)	14 (13.6)	26 (25.2)
Student (including language courses)	81 (39.3)	46 (44.7)	35 (34.0)
Other	10 (4.9)	5 (4.9)	5 (4.9)
Refugee status, n (%)			
Asylum procedure ongoing	16 (7.8)	10 (8.7)	6 (5.8)
Resident permit	150 (72.8)	71 (68.9)	79 (76.7)
Dutch citizenship	26 (12.6)	13 (12.6)	13 (12.6)
Other	2 (1.0)	2 (1.9)	0
Missing	12 (5.8)	7 (6.8)	5 (4.9)
Time elapsed (months) since arriving in the Netherlands*, mean (SD) (range)	44.07 (23.07) (1–113)	42.22 (23.57) (1–97)	45.94 (22.53) (2–113)
Educational level, n (%)			
No education	1 (0.5)	0	1 (1.0)
Basic education	29 (14.1)	10 (9.7)	19 (18.4)
Technical/vocational secondary	6 (2.9)	3 (2.9)	3 (2.9)
Technical diploma	13 (6.3)	7 (6.8)	6 (5.8)
Certificate of associate degree	18 (8.7)	11 (10.7)	7 (6.8)
General secondary education	37 (18.0)	21 (20.4)	16 (15.5)
Bachelor	82 (39.8)	41 (39.8)	41 (39.8)
Master	20 (9.7)	10 (9.7)	10 (9.7)
PhD	0	0	0
Depression and anxiety (HSCL-25 total)	2.36 (0.62)	2.31 (0.64)	2.41 (0.61)
Depression (HSCL-25 subscale), mean (SD)	2.43 (0.71)	2.47 (0.72)	2.38 (0.70)
Probable depression, n (%)†	142 (68.9)	66 (64.1)	76 (73.8)
Anxiety (HSCL-25 subscale), mean (SD)	2.20 (0.64)	2.16 (0.66)	2.24 (0.61)
Probable anxiety, n (%)‡	129 (62.6)	55 (53.4)	74 (71.8)
PTSD symptoms (PCL-5), mean (SD)	34.35 (16.89)	33.13 (17.76)	35.57 (15.96)
Probable PTSD, n (%)§	109 (52.9)	52 (50.5)	57 (55.3)
Functional impairment (WHODAS 2.0), mean (SD)	29.46 (7.72)	29.09 (8.07)	29.84 (7.39)
Self-identified problems (PSYCHLOPS), mean (SD)	15.42 (3.71)	15.25 (3.74)	15.58 (3.69)
No of traumatic events, mean (SD) (range)	9.60 (5.08) (0–26)	9.90 (5.52) (0–26)	9.30 (4.61) (0–21)
PMLD, mean (SD) (range)	6.95 (3.55) (0–16)	6.74 (3.59) (0–16)	7.17 (3.51) (0–15)

*n=200.

†Based on HSCL-25 depression subscale cut-off ≥2.10.

‡Based on HSCL-25 anxiety subscale cut-off ≥2.00.

§Based on PCL-5 ≥33.

CAU, care as usual; HSCL, Hopkins Symptom Checklist; PCL-5, PTSD Checklist for DSM-5; PM+, Problem Management Plus; PM+/CAU, PM+ in addition to care as usual; PMLD, post-migration living difficulties ; PSYCHLOPS, Psychological Outcomes Profiles; PTSD, post-traumatic stress disorder; WHODAS 2.0, WHO Disability Assessment Schedule 2.0.

Retention at 3-month follow-up was 85.4%, with data available for 84 participants (81.5%) in PM+/CAU and 92 (89.3%) in CAU. Participants lost at 3-month follow-up versus those retained did not differ in terms of baseline characteristics (online supplemental table S1). At 3-month follow-up, masking was successful for 144 (81.8%) participants.

10.1136/bmjment-2022-300637.supp2 Supplementary data

In PM+/CAU, 87 participants (84.5%) attended a minimum of four PM+ sessions (see figure 1). Of those attending at least one session, 64 (62.8%) attended in-person, 25 (24.5%) online (ie, video calls) and 13 (12.7%) in-person and online (ie, hybrid). PM+ helper checklists indicated 97.5% of the protocol was carried out. Thirty-six participants (35.3%) provided IC for audio recordings. Independent ratings (3/50 tapes were excluded due to technical problems; inter-rater reliability Cohen’s *κ*=0.91) indicated on average 77.4% of the protocol was delivered adequately.

### Primary outcome

LMMs (see table 2) showed an overall positive intervention effect. Condition had a significant moderate effect on HSCL-25 depression/anxiety total score over time adjusted for baseline, with lower scores for PM+/CAU relative to CAU. At 1-week postassessment, the estimated marginal mean was 1.95 for PM+/CAU and 2.27 for CAU, giving a mean difference of −0.32 (95% CI −0.450 to −0.191; p<0.0001, Cohen’s *d*=0.50). At 3-month follow-up, the estimated marginal mean was 1.94 for PM+/CAU and 2.19 for CAU, giving a mean difference of −0.25 (95% CI −0.385 to −0.122; p=0.0001, Cohen’s *d*=0.41). Similar effects were found for the HSCL-25 depression and anxiety subscales at 1-week postassessment (depression: −0.34; 95% CI −0.486 to −0.199; p<0.0001, Cohen’s *d*=0.50; anxiety: −0.29; 95% CI −0.430 to −0.155; p<0.0001, Cohen’s *d*=0.46) and at 3-month follow-up (depression: −0.28; 95% CI −0.421 to −0.131; p=0.0002, Cohen’s *d*=0.42; anxiety −0.23; 95% CI −0.365 to −0.087; p=0.001, Cohen’s *d*=0.35).

**Table 2:** Summary statistics and results from mixed-model analysis of primary and secondary outcomes

Outcome	Time point	Descriptive statistics, mean (SD)				Mixed-model analysis			Covariate-adjusted mixed-model analysis*		
		N	PM+/CAU (n=103)	N	CAU (n=103)	Difference in LS mean (95% CI)	P value	Effect size†	Difference in LS mean (95% CI)	P value	Effect size†
**Primary outcome**											
HSCL-25 total	Baseline	103	2.31 (0.64)	103	2.41 (0.61)						
	Overall effect‡					−0.29 (−0.397 to −0.179)	<0.0001	0.46	−0.24 (−0.219 to −0.070)	<0.0001	0.38
	Postassessment	85	1.91 (0.61)	93	2.31 (0.66)	−0.32 (−0.450 to −0.191)	<0.0001	0.50	−0.27 (−0.392 to −0.154)	<0.0001	0.43
	3-month follow-up	82	1.88 (0.61)	91	2.23 (0.63)	−0.25 (−0.385 to −0.122)	0.0001	0.41	−0.21 (−0.326 to −0.085)	0.0009	0.33
HSCL-25 depression	Baseline	103	2.39 (0.69)	103	2.47 (0.72)						
	Overall effect‡					−0.31 (−0.430 to −0.190)	<0.0001	0.46	−0.26 (−0.369 to −0.155)	<0.0001	0.39
	Postassessment	85	1.92 (0.64)	93	2.33 (0.77)	−0.34 (−0.486 to −0.199)	<0.0001	0.50	−0.23 (−0.427 to −0.163)	<0.0001	0.43
	3-month follow-up	82	1.91 (0.63)	91	2.28 (0.69)	−0.28 (−0.421 to −0.131)	0.0002	0.42	−0.23 (−0.363 to −0.095)	0.0009	0.36
HSCL-25 anxiety	Baseline	103	2.16 (0.66)	103	2.24 (0.61)						
	Overall effect‡					−0.26 (−0.375 to −0.145)	<0.0001	0.41	−0.21 (−0.308 to −0.102)	0.0001	0.41
	Postassessment	85	1.85 (0.65)	93	2.21 (0.64)	−0.29 (−0.430 to −0.155)	<0.0001	0.46	−0.24 (−0.367 to −0.112)	0.0002	0.37
	3-month follow-up	82	1.84 (0.64)	91	2.15 (0.64)	−0.23 (−0.365 to −0.087)	0.001	0.35	−0.17 (−0.299 to −0.041)	0.01	0.27
**Secondary outcomes**											
PCL-5	Baseline	103	33.22 (17.84)	103	35.57 (15.96)						
	Overall effect‡					−6.70 (−9.730 to −3.675)	<0.0001	0.40	−5.51 (−8.911 to −4.764)	<0.0001	0.33
	Postassessment	85	20.89 (17.49)	92	28.76 (16.52)	−6.92 (−10.546 to −3.291)	0.0002	0.41	−5.81 (−9.064 to −2.503)	0.0006	0.34
	3-month follow-up	82	19.79 (16.59)	92	28.21 (16.38)	−6.49 (−10.150 to −2.834)	0.0005	0.39	−5.21 (−8.491 to −1.888)	0.002	0.32
WHODAS 2.0	Baseline	103	29.09 (8.07)	103	29.84 (7.38)						
	Overall effect‡					−1.72 (−3.241 to −0.196)	0.02	0.21	−1.04 (−2.446 to −0.392)	0.15	0.13
	Postassessment	85	24.76 (8.51)	93	26.90 (7.90)	−1.81 (−3.644 to −0.018)	0.05	0.22	−1.12 (−2.855 to −0.651)	0.22	0.14
	3-month follow-up	82	23.40 (8.25)	92	25.88 (7.38)	−1.64 (−3.489 to −0.214)	0.08	0.21	−0.98 (−2.739 to −0.797)	0.28	0.12
PSYCHLOPS	Baseline	103	15.38 (3.71)	103	15.72 (3.43)						
	Overall effect‡					−1.80 (−2.768 to −0.831)	0.0002	0.37	−1.42 (−2.323 to −0.523)	0.002	0.29
	Postassessment	85	11.44 (4.78)	92	13.86 (4.58)	−2.23 (−3.441 to −1.023)	0.0003	0.48	−1.87 (−3.028 to −0.711)	0.001	0.40
	3-month follow-up	84	10.56 (5.38)	91	12.25 (4.74)	−1.34 (−2.561 to −0.127)	0.03	0.26	−0.97 (−2.138 to −0.184)	0.10	0.19

*Covariates included in these models are gender; age; education; marital status; work status; trauma; post-migration living difficulties; and probable depression, anxiety and PTSD measured at baseline.

†Effect sizes were calculated using the difference in least square means between the PM+/CAU and CAU group divided by the pooled SD at that assessment.

‡This is the overall effect of condition on average over the two follow-up assessments.

CAU, care as usual; HSCL, Hopkins Symptom Checklist; LS, least squares; PCL-5, PTSD Checklist for DSM-5; PM+, Problem Management Plus; PM+/CAU, PM+ in addition to care as usual; PSYCHLOPS, Psychological Outcomes Profiles; PTSD, post-traumatic stress disorder; WHODAS 2.0, WHO Disability Assessment Schedule 2.0.

### Secondary outcomes

At 3-month follow-up, condition had a significant small-to-moderate effect on PCL-5, with lower scores for PM+/CAU relative to CAU (−6.49; 95% CI −10.150 to −2.834, p=0.0005, Cohen’s *d*=0.39), and a significant small effect on PSYCHLOPS, with lower scores for PM+/CAU versus CAU (−1.34; 95% CI −2.561 to −0.127; p=0.03, Cohen’s *d*=0.26). For WHODAS 2.0, condition was not significant 3 months after the intervention (−1.64; 95% CI −3.489 to −0.214; p=0.08, Cohen’s *d*=0.21), although there was a small average effect of condition over the follow-up assessments together (1-week and 3-month follow-up) in favour of PM+/CAU (−1.72; 95% CI −3.241 to −0.220; p=0.02, Cohen’s *d*=0.21).

Covariate-adjusted LMMs (including all covariates) were consistent with the primary LMM but with overall smaller effect sizes (table 2).

Moderation analyses of the primary outcome (HSCL-25 total) showed that intervention effects were larger for participants with a higher educational background at 1-week follow-up (p=0.04) and at 3-month follow-up (p=0.02) and for participants who scored above cut-off at baseline for depression (p<0.0001 and p<0.0001, respectively), anxiety (p=0.0009 and p=0.002, respectively) and PTSD (p<0.0001 and p<0.0001, respectively). Participants with more post-migration living difficulties at 3-month follow-up benefited less from PM+ at 3-month follow-up (1-week follow-up: p=0.49; 3-month follow-up: p=0.04). Other variables (ie, gender, age, marital status, work status, traumatic events and post-migration living difficulties at baseline) were not found to be significant effect modifiers.

Sensitivity analyses focusing on participants retained at 3-month follow-up and per protocol were consistent with the primary analysis (see online supplemental tables S2 and S3). Sensitivity analysis of the PM+ delivery formats, a deviation from the study protocol due to COVID-19 restrictions, showed that participants receiving in-person sessions (n=64) had significantly lower HSCL-25 total scores relative to CAU at 1-week (−0.39; 95% CI −0.544 to −0.244; p<0.0001, Cohen’s *d*=0.61) and 3-month follow-up (−0.34; 95% CI −0.492 to −0.188; p<0.0001, Cohen’s *d*=0.54). Participants receiving online/hybrid sessions (n=38) also had significantly lower HSCL-25 total scores relative to CAU at 1 week (−0.21; 95% CI −0.383 to −0.042; p=0.01, Cohen’s *d*=0.33) but not at 3-month follow-up (−0.13; 95% CI −0.301 to 0.042; p=0.14, Cohen’s *d*=0.21) (online supplemental table S4).

10.1136/bmjment-2022-300637.supp3 Supplementary data

10.1136/bmjment-2022-300637.supp4 Supplementary data

10.1136/bmjment-2022-300637.supp5 Supplementary data

At 3-month follow-up, 34 PM+/CAU participants had a reliable decrease in HSCL-25 total scores, of whom 2 had a clinically significant change (ie, recovered). In CAU, 22 participants had a reliable decrease in HSCL-25 scores, of whom none recovered. Three months after the intervention, two participants in PM+/CAU versus five participants in CAU had a reliable increase in HSCL-25 scores (ie, deteriorated) (table 3). We estimated a number needed to treat of 4.2 for depression (risk difference=−0.24; 95% CI −0.314 to −0.166) and of 8.2 for anxiety (risk difference=−0.12; 95% CI −0.020 to −0.043).

**Table 3:** Reliable change index at postassessment and 3-month follow-up for the HSCL-25 (completers only)

RCI	1-week postassessment		3-month follow-up	
	PM+/CAU (n=85)	CAU (n=93)	PM+/CAU (n=84)	CAU (n=92)
Recovered, n (%)*	0	1 (1.1)	2 (2.4)	0
Improved without recovery, n (%)†	35 (41.2)	15 (16.1)	32 (38.1)	22 (23.9)
Deteriorated, n (%)†	4 (4.7)	9 (9.7)	2 (2.4)	5 (5.4)
No change, n (%)	46 (54.1)	68 (73.1)	48 (57.1)	65 (70.7)

Recovered=clinical significant reliable change; improved without recovery=no clinical significant reliable change; deteriorated=reliable change with worsening of symptoms; no change=no reliable change.

*The Clinical Significant Change cut-off for the HSCL-25 (total scale) was calculated by subtracting 2 SD of the baseline mean for the full sample.

†The RCI for the HSCL-25 (total score) was calculated using the baseline SD for the full sample and baseline Cronbach’s α as test–retest reliability coefficient^27^.

RCI, reliable change index.

Four SAEs were reported (two in each group, PM+/CAU: both hospitalised due to medical illness, CAU: one suicide attempt and one hospitalised due to medical illness), but all were assessed as unlikely to be related to the intervention or trial procedures.

## Discussion

This study evaluated a brief, behavioural intervention (PM+) for Syrian refugees with elevated levels of psychological distress in the Netherlands. Our main finding was that PM+ delivered by Syrian non-specialist helpers reduced symptoms of depression, anxiety, PTSD and self-identified problems 3 months later. Furthermore, our study has shown that PM+ is safe and not associated with any adverse outcomes.

Our key findings on depression and anxiety are consistent with earlier studies on individual PM+ in non-refugee samples in low-resource settings.^10 11^ The current study was the first to demonstrate that individual PM+ is also effective for refugees in a high-income setting. Although larger effect sizes are reported for psychotherapy in LMICs versus HICs,^28^ our findings on depression are consistent with the treatment effect of task-sharing interventions for depression in LMICs^13 29^ and of psychotherapies for depression compared with care-as-usual control groups in HICs.^28^ The magnitude of effect for anxiety was not as strong as what we found for depression. This is also reflected in the larger number needed to treat for anxiety (8.2) in comparison with depression (4.2) and is in line with previous PM+ trials that found relatively smaller effects on anxiety compared with depression^10 30^ or no effect on anxiety.^13^ It thus seems that PM+ strategies better address depressive symptoms, for example through re-engagement with pleasant activities as a result of behavioural activation.

Another key finding is that, in light of (accumulated) trauma and ongoing stressors typically faced by refugees, PM+ led to reductions in symptoms of PTSD. About half of the participants in the current sample had scores signalling elevated PTSD symptomatology, and our study findings suggest that individuals with a probable PTSD diagnosis may benefit even more from the intervention. This is surprising since PM+ does not include exposure to a traumatic memory, which is assumed to be a core component of effective treatments for PTSD.^31^ Previous studies with individual PM+^10 11^ similarly reported benefits in improving PTSD, whereas this was not found for the group version.^13 30 32^ A possible explanation is that PM+, particularly when delivered individually, may provide space for discussing personal events and experiences and may as such enable the emotional processing of personal traumatic memories or address individual avoidance behaviour during the sessions.

Although we did not find a significant effect of PM+ on functional impairment 3 months after the intervention, our study demonstrated a significant average effect for post- and 3-month follow-up together. Previous studies with individual and group PM+ reported mixed results on functional impairment.^10 11 13 30^ Impairment and restrictions related to the COVID-19 pandemic that started amidst the trial may have affected participants’ daily functioning and impeded potential benefits of PM+. Although it might be that the pandemic has had negative impacts on intervention effectiveness, it has also shown that the intervention is adaptable to changing circumstances and has the potential to be delivered online. Perhaps unsurprising given the context in which the format was rapidly adjusted, the effects of online/hybrid PM+ delivery were smaller in magnitude compared with in-person sessions. These analyses were, however, of exploratory nature and results should be interpreted with caution.

Higher education was associated with greater treatment effects. It might be that higher educated individuals are more likely to make better use of PM+ skills.^32^ We also found that treatment effects were smaller for individuals reporting more post-migration living difficulties during the trial, suggesting that individuals with many ongoing stressors and insecurity might benefit less from the intervention.^32^ Our study was, however, underpowered for moderation analyses, so these findings should be cautiously interpreted, as are our findings that baseline scores above the clinical cut-off were associated with larger treatment effects. In this regard, further analysis using individual participant data of PM+ trials will allow for more sophisticated modelling of effect modifiers.

Strengths of this study include good retention of participants at follow-up (85%) (compared to attrition rates of 85% and 66% in RCTs evaluating a task-shared psychological intervention in refugees in community settings in Türkiye and Western Europe),^33 34^ feasibility of training of refugee non-specialist helpers and successful delivery of the intervention and trial procedures during the COVID-19 pandemic. This study also has a number of limitations. First, our sample predominantly included Syrians with a residence permit. We cannot assume the intervention is similarly effective in refugees experiencing uncertainty about their asylum status, which is a main source of distress for asylum seekers.^35^ Furthermore, the educational level in the sample was relatively high, hindering generalisation to refugees with a lower educational background. Second, PM+ session delivery shifted from in-person to online/hybrid sessions due to COVID-19 restrictive measures. Study effects may have been affected by this unplanned change in delivery format. Third, mental healthcare utilisation among refugees is typically low and so for most control participants CAU was not an active control condition.

Adding PM+ to the array of available social (eg, community support, social benefit/welfare and housing) and mental health and psychosocial support services in the Netherlands may improve mental health and well-being for underserved populations like refugees. Beyond effectiveness, it is important to determine whether the intervention is cost-effective in a HIC.^15 16^ We are conducting an economic evaluation to assess cost-effectiveness and explore whether PM+ has the potential for being integrated with the Dutch healthcare system, for example as a first step in ‘stepped-care’. The responsiveness of health systems to refugees in HICs (compared with LMICs neighbouring Syria) is typically reduced by cultural and language barriers, and PM+ delivered by peers may offer an opportunity to overcome these barriers. Scale-up in a HIC such as the Netherlands may require political, regulatory and health system changes, including sustainable financing, policies that enable non-specialist helpers as providers, the establishment of a resource and knowledge centre to support delivery and quality of the intervention, and resources to identify potential service users.^36^ This is an important step, especially given the steep rise of refugees in Europe since the outbreak of war in Ukraine.^1^ Peer-provided interventions such as PM+ may enhance responsiveness of health systems to refugees from various countries.

PM+ delivered by peer providers is an effective intervention to reduce symptoms of depression, anxiety and PTSD, as well as self-identified problems in Syrian refugees. This study is the first RCT on PM+ for refugees in a HIC and suggests that PM+ may be of potential utility in a setting where access to specialist services is typically hampered by waitlists and communication difficulties. Further research may evaluate the intervention’s long-term effectiveness and the potential for scale-up.

## Data Availability

Data are available on reasonable request. The Vrije Universiteit Amsterdam (VU) will keep a central data repository of all data collected in the STRENGTHS project. The data will be available on reasonable request to the STRENGTHS consortium. Data access might not be granted to third parties when this would interfere with relevant data protection and legislation in the countries participating in this project and any applicable European Union legislation regarding data protection. The PM+ training manual and intervention manual are available through the consortium. Interested researchers can contact prof. dr. Marit Sijbrandij at e.m.sijbrandij@vu.nl to initiate the process.
