# Supplementary material for: Peer-provided psychological intervention for Syrian refugees: results of a randomised controlled trial on the effectiveness of Problem Management Plus
Source: BMJ Ment Health. 2023 Feb 8;26(1):e300637. doi: 10.1136/bmjment-2022-300637 (PMC10035776; doi:10.1136/bmjment-2022-300637)
Supplement: Supplementary data [file bmjment-2022-300637supp002.pdf]

Table S1.
Baseline Characteristics of Participants Retained and Lost at Follow-Up

	Retained at 3-month follow-up (*n*=176)	Lost at 3-month follow-up (*n*=30)	t/χ^2	*p*
Sex, *n* of men (%)	105 (59.7%)	22 (73.3%)	2.027	0.15
Age, *M* (*SD*) [range]	36.98 (11.56) [18-69]	33.80 (12.44) [20-62]	1.378	0.17
Marital status, *n* (%)			6.505	0.26
Never married	12 (40.0%)	58 (33.0%)		
Currently married	12 (40.0%)	87 (49.4%)		
Separated	2 (6.7%)	2 (1.1%)		
Divorced	4 (13.3%)	20 (11.4%)		
Widowed	0	5 (2.8%)		
Cohabiting	0	4 (2.3%)		
Work status			7.570	0.47
Paid work	32 (18.2%)	4 (13.3%)		
Non-paid work	27 (15.3%)	3 (10.0%)		
Keeping house	4 (2.3%)	3 (10.0%)		
Retired	2 (1.1%)	0		
Unemployed	33 (18.8%)	7 (23.3%)		
Student	70 (39.8%)	11 (36.7%)		
Other	8 (4.5%)	2 (6.7%)		
Refugee status, *n* (%)			6.460	0.09
Asylum procedure ongoing	11 (6.3%)	5 (16.7%)		
Resident permit	129 (73.3%)	21 (70.0%)		
Dutch citizenship	24 (13.6%)	2 (6.7%)		
Other	1 (0.6%)	1 (3.3%)		
Missing	10 (6.3%)	1 (3.3%)		
Time elapsed (months) since arriving in the Netherlands,[a] *M* (*SD*) [range]	44.40 (22.67) [1-97]	42.13 (25.61) [2-113]	0.497	0.62
Educational level, *n* (%)			2.334	0.31
No/basic education	24 (13.6%)	6 (20.0%)		
Secondary education	61 (34.7%)	13 (43.3%)		
Tertiary education	91 (51.7%)	11 (36.7%)		
Depression and anxiety (HSCL-25 total)	2.35 (.64)	2.43 (.50)	-0.677	0.49
PTSD symptoms (PCL-5), *M* (*SD*)	33.76 (16.91)	38.13 (16.83)	-1.310	0.19
Functional impairment (WHODAS 2.0), *M* (*SD*)	29.36 (7.73)	30.07 (7.81)	-0.464	0.32
Self-identified problems (PSYCHLOPS), *M* (*SD*)	15.59 (3.53)	15.30 (3.81)	0.412	0.34
Number of traumatic events, *M* (SD) [range]	9.76 (5.11) [0-26]	8.70 (4.97) [1-18]	1.056	0.29
PMLD, *M* (SD) [range]	6.81 (3.55) [0-16]	7.76 (3.51) [2-15]	-1.364	0.17

[a] *n*=200; PMLD = post-migration living difficulties; PTSD = posttraumatic stress disorder
