# Supplementary material for: Peer-provided psychological intervention for Syrian refugees: results of a randomised controlled trial on the effectiveness of Problem Management Plus
Source: BMJ Ment Health. 2023 Feb 8;26(1):e300637. doi: 10.1136/bmjment-2022-300637 (PMC10035776; doi:10.1136/bmjment-2022-300637)
Supplement: Supplementary data [file bmjment-2022-300637supp003.pdf]

Table S2.

Summary Statistics and Results from Mixed-Model Analysis of Primary and Secondary Outcomes for Participants Retained at 3-Month Follow-Up

		Descriptive statistics, *M* (*SD*)				Mixed-model analysis		
Outcome	Time point	*N*	PM+/CAU (*n*=103)	*N*	CAU (*n*=103)	Difference in Least Squares mean (95% CI)	*p*-value	Effect size [b]
Primary outcome								
HSCL-25 Total	Baseline	84	2.29 (0.66)	92	2.40 (0.63)			
	Overall effect [a]					-0.291 (-0.404, -0.179)	<0.0001	0.46
	Post-assessment	78	1.88 (0.59)	87	2.30 (0.67)	-0.329 (-0.464, -0.194)	<0.0001	0.52
	3-months follow-up	82	1.88 (0.61)	91	2.23 (0.63)	-0.257 (-0.389, -0.124)	0.0001	0.41
HSCL-25 Depression	Baseline	84	2.39 (0.72)	92	2.51 (0.70)			
	Overall effect [a]					-0.313 (-0.437, -0.189)	<0.0001	0.47
	Post-assessment	78	1.93 (0.62)	87	2.37 (0.74)	-0.350 (-0.499, -0.200)	<0.0001	0.51
	3-months follow-up	82	1.91 (0.63)	91	2.28 (0.69)	-0.279 (-0.425, -0.132)	0.0002	0.42
HSCL-25 Anxiety	Baseline	84	2.13 (0.65)	92	2.25 (0.64)			
	Overall effect [a]					-0.267 (-0.385, -0.148)	<0.0001	0.42
	Post-assessment	78	1.82 (0.62)	87	2.37 (0.74)	-0.306 (-0.448, -0.162)	<0.0001	0.48
	3-months follow-up	82	1.84 (0.64)	91	2.15 (0.64)	-0.231 (-0.370, -0.090)	0.001	0.36
Secondary outcomes								
PCL-5	Baseline	84	32.48 (17.73)	92	34.94 (16.13)			
	Overall effect [a]					-6.79 (-9.906, -3.682)	<0.0001	0.41
	Post-assessment	78	20.09 (17.01)	87	29.06 (16.77)	-7.05 (-10.827, -3.281)	0.0002	0.42
	3-months follow-up	82	19.79 (16.59)	92	25.88 (7.38)	-6.56 (-10.246, -2.866)	0.0005	0.40
WHODAS 2.0	Baseline	84	28.83 (8.08)	92	29.84 (7.40)			
	Overall effect [a]					-1.83 (-3.406, -0.259)	0.02	0.23
	Post-assessment	78	24.45 (8.07)	87	27.00 (8.06)	-1.97 (-3.887, -0.049)	0.04	0.24
	3-months follow-up	82	23.40 (8.25)	92	25.88 (7.38)	-1.70 (-3.582, 0.171)	0.07	0.22
PSYCHLOPS	Baseline	84	15.17 (3.90)	92	15.98 (3.13)			
	Overall effect [a]					-1.90 (-2.901, -0.905)	<0.0001	0.39
	Post-assessment	78	11.36 (4.82)	86	14.09 (4.37)	-2.42 (-3.683, -1.152)	0.0002	0.53
	3-months follow-up	84	10.56 (5.38)	91	12.25 (4.74)	-1.40 (-2.624, -0.170)	0.02	0.28

[a] This is the overall effect of condition on average over the two follow-up assessments; [b] Effect sizes were calculated using the difference in least square means between the PM+/CAU and CAU group divided by the pooled *SD* at that assessment.
