# Supplementary material for: Peer-provided psychological intervention for Syrian refugees: results of a randomised controlled trial on the effectiveness of Problem Management Plus
Source: BMJ Ment Health. 2023 Feb 8;26(1):e300637. doi: 10.1136/bmjment-2022-300637 (PMC10035776; doi:10.1136/bmjment-2022-300637)
Supplement: Supplementary data [file bmjment-2022-300637supp004.pdf]

Table S3.
*Summary Statistics and Results from Mixed-Model Analysis of Primary and Secondary Outcomes for Per Protocol Sample*

| | | Descriptive statistics, *M* (*SD*) | | | | Mixed-model analysis | | |
|---|---|---|---|---|---|---|---|---|
| Outcome | Time point | *N* | PM+/CAU (*n*=103) | *N* | CAU (*n*=103) | Difference in Least Squares mean (95% CI) | *p*-value | Effect size [b] |
| **Primary outcome** | | | | | | | | |
| HSCL-25 Total | Baseline | 87 | 2.29 (0.67) | 103 | 2.41 (0.61) | | | |
| | Overall effect [a] | | | | | -0.293 (-0.403, -0.182) | <0.0001 | 0.46 |
| | Post-assessment | 82 | 1.91 (0.61) | 93 | 2.31 (0.66) | -0.327 (-0.458, -0.196) | <0.0001 | 0.51 |
| | 3-months follow-up | 79 | 1.88 (0.62) | 91 | 2.23 (0.63) | -0.258 (-0.390, -0.125) | <0.0001 | 0.41 |
| HSCL-25 Depression | Baseline | 87 | 2.40 (0.73) | 103 | 2.52 (0.69) | | | |
| | Overall effect [a] | | | | | -0.312 (-0.434, -0.190) | <0.0001 | 0.46 |
| | Post-assessment | 82 | 1.96 (0.63) | 93 | 2.37 (0.73) | -0.345 (-0.490, -0.200) | <0.0001 | 0.50 |
| | 3-months follow-up | 79 | 1.91 (0.63) | 92 | 2.28 (0.69) | 0.278 (-0.425, -0.131) | 0.0002 | 0.42 |
| HSCL-25 Anxiety | Baseline | 87 | 2.13 (0.67) | 103 | 2.24 (0.61) | | | |
| | Overall effect [a] | | | | | -0.270 (-0.386, -0.153) | <0.0001 | 0.42 |
| | Post-assessment | 82 | 1.85 (0.66) | 93 | 2.21 (0.64) | -0.305 (-0.444, -0.166) | <0.0001 | 0.47 |
| | 3-months follow-up | 79 | 1.83 (0.64) | 91 | 2.15 (0.64) | -0.234 (-0.374, -0.093) | 0.001 | 0.36 |
| **Secondary outcomes** | | | | | | | | |
| PCL-5 | Baseline | 87 | 32.90 (18.40) | 103 | 35.57 (15.96) | | | |
| | Overall effect [a] | | | | | -6.699 (-9.761, -3.639) | <0.0001 | 0.40 |
| | Post-assessment | 82 | 21.04 (17.56) | 92 | 28.80 (16.54) | -6.778 (-10.438, -3.119) | <0.0001 | 0.40 |
| | 3-months follow-up | 79 | 19.76 (16.72) | 92 | 28.22 (16.38) | -6.619 (-10.312, -2.929 | <0.0001 | 0.40 |
| WHODAS 2.0 | Baseline | 87 | 29.11 (8.27) | 103 | 29.84 (7.38) | | | |
| | Overall effect [a] | | | | | -1.530 (-3.072, 0.0106) | 0.05 | 0.19 |
| | Post-assessment | 82 | 24.95 (8.60) | 93 | 26.90 (7.90) | -1.652 (-3.509, 0.205) | 0.08 | 0.20 |
| | 3-months follow-up | 79 | 23.62 (8.30) | 92 | 25.88 (7.38) | -1.406 (-3.285, 0.472) | 0.14 | 0.18 |
| PSYCHLOPS | Baseline | 87 | 15.17 (3.92) | 103 | 15.72 (3.43) | | | |
| | Overall effect [a] | | | | | -1.740 (-2.723, -0.756) | <0.0001 | 0.35 |
| | Post-assessment | 82 | 11.55 (4.76) | 92 | 13.86 (4.58) | -2.136 (-3.367, -0.904) | <0.0001 | 0.46 |
| | 3-months follow-up | 80 | 10.51 (5.43) | 91 | 12.25 (4.74) | -1.334 (-2.578, -0.089) | 0.03 | 0.26 |

[a] This is the overall effect of condition on average over the two follow-up assessments; [b] Effect sizes were calculated using the difference in least square means between the PM+/CAU and CAU group divided by the pooled *SD* at that assessment.
