# Supplementary material for: Peer-provided psychological intervention for Syrian refugees: results of a randomised controlled trial on the effectiveness of Problem Management Plus
Source: BMJ Ment Health. 2023 Feb 8;26(1):e300637. doi: 10.1136/bmjment-2022-300637 (PMC10035776; doi:10.1136/bmjment-2022-300637)
Supplement: Supplementary data [file bmjment-2022-300637supp005.pdf]

Table S4.

Summary Statistics and Results from Mixed-Model Analysis of Primary Outcome for Different PM+ Delivery Formats

		Descriptive statistics, *M* (*SD*)						Mixed-model analysis					
		PM+/CAU				CAU		Difference in Least Squares mean (95% CI)					
Outcome	Time point	*N*	In-person sessions (*n*=64)	*N*	Video/hybrid sessions (*n*=38)	*N*	CAU (*n*=103)	PM+ in-person sessions vs CAU	*p*-value	Effect size [b]	PM+ video/hybrid sessions vs CAU	*p*-value	Effect size [b]
HSCL-25	Baseline	64	2.41 (0.64)	38	2.14 (0.60)	103	2.41 (0.61)						
	Overall effect [a]							-0.37 (-0.493, -0.241)	<0.0001	0.58	-0.17 (-0.315, -0.028)	0.01	0.28
	Post-assessment	50	1.91 (0.63)	35	1.91 (0.59)	93	2.31 (0.66)	-0.39 (-0.544, -0.244)	<0.0001	0.61	-0.21 (-0.383, -0.042)	0.01	0.33
	3-months follow-up	48	1.86 (0.62)	34	1.90 (0.60)	91	2.23 (0.63)	-0.34 (-0.492, -0.188)	<0.0001	0.54	-0.13 (-0.301, 0.042)	0.14	0.21

[a] This is the overall effect of condition on average over the two follow-up assessments; [b] Effect sizes were calculated using the difference in least square means between the PM+/CAU and CAU group divided by the pooled *SD* at that assessment.
